# Acute Psoas Abscess Discharged through an Opening into the Ascending Colon

**Published:** 1888-08

**Authors:** J. McFadden Gaston

**Affiliations:** Professor of Surgery, Southern Medical College, Atlanta, Georgia


### Southern Medical Record.

A MONTHLY JOURNAL OF PRACTICAL MEDICINE.

☞All Communications must be addressed to R. C. WORD, M. D., Managing Editor.

"*Quicquid Præcipies Esto Brevis.*"

VOL. XVIII. ATLANTA, GA., AUGUST, 1888. No. 8.

### ORIGINAL AND SELECTED ARTICLES.

### ACUTE PSOAS ABSCESS DISCHARGED THROUGH AN OPENING INTO THE ASCENDING COLON.

BY J. MCFADDEN GASTON, M. D.,
Professor of Surgery, Southern Medical College, Atlanta, Georgia.

On the 25th June, after retiring for the night, I was called by telephone to visit a patient in consultation with Dr. W. S. Edwards. He was under the impression that the symptoms indicated the development of an abscess, and hence had advised calling surgical aid in the management of the case.

The following history of the preliminary disorder and treatment has been kindly given by him; and I avail myself also of his statements in regard to the effects of the measures adopted in accordance with this conference, and our subsequent consultation on June 29th.

STATEMENT OF W. S. EDWARDS, M. D.

I saw a gentleman about thirty-two years old for the first time on the 21st day of June, 1888. He complained of a peculiar feeling with a dull heavy pain in and around the stomach. His bowels were constipated, had not moved in thirty-six hours; tongue coated; and appetite very poor. I gave him a preparation of—

℞. Quinia sulp....................................grs. xxxv,
Hydr. chlo. mit..................................grs. v,

M. Et ft. caps. No. 10. Sig.—one every three hours.

I saw him on the following morning and the medicine had failed to move the bowels. At this time he was very much nauseated. I gave him—

℞. Sulph. magn ℥ i,

Tr. opii deod ʒ ss,

Sy. aurenti ℥ ii,

Aqua, q. s ℥ iv.

M. Et. ft. sol. Half at once, remainder in three hours. The medicine had desired effect, and he felt much relieved for the time being; but as soon as the opium lost its effect, he had more severe pain than before, but it had changed its location, it was lower down now, just to the left of the umbilicus, but the pain over the stomach had disappeared, and he could bear pressure now over that point that he could not before. Now he could not endure any pressure on the left side of the abdomen, but the right side was unaffected by pressure. He remained in this condition, with the pain very severe unless anodynes were freely used, till the morning of the 23rd of June, when his bowels began to move too frequently—four to six copious actions in six hours. I relieved this trouble promptly with astringents. Up to this time his temperature had never been over 101½°, circulation never over 100. On the evening of the 23rd he desired to change his room, for private reasons, and in doing so had to walk about thirty steps and remain in the elevator about two minutes. I took his temperature just before he moved, it was 101¼°, and in less than half an hour after the move his temperature was 104½°; circulation 140; extremities grew exceedingly cold. Hot bottle and friction relieved this, and his temperature came down to 102°; circulation 120. At this point there was another decided change in the location of the pain. I saw him at ten o'clock on the night of the 24th, it had moved from the left side of the abdomen entirely, and he could bear firm pressure anywhere on that side, but the pain was on the right side extending from the inguinal region around toward the right kidney, the most sensitive point being about five or six inches to the right of the kidney, where it remained with his temperature steadily increasing till it reached 103¾°. The following night his pains were so severe that I called Dr. Gaston in consultation. After a thorough investigation Dr. Gaston expressed his opinion, saying he thought we had a local inflammation, involving the right lumbar region, but without any definite location. We gave him hydro chlo. mit. and bicarb. soda, followed the next morning by spts. terbinth, ʒ j; oleum ricini, ℥ j. About 3 p. m. he had a large action, and to relieve the febrile condition gave sulph. quinine and camphorated

Dover's powders, which counteracted this condition at once; but as he required an anodyne, we kept it up for several days, and to counteract the effect of the opium upon the bowels, I gave him oleum ricini on the following days up to the 29th, at which time his bowels moved of their own accord, and were very regular. We began the use of flax seed poultice, and a camphorated belladonna ointment over the lumbar region; and with modifications to be stated by Dr. Gaston, local applications were continued till July 6. But I only desire to give the history of this case up to June 29, where Dr. Gaston will take it up and give the history, diagnosis and treatment from then till the 6th of July. I saw the patient, however, every day during this time, and am satisfied that we had a well developed psoas abscess. But as this comes under the head of surgery, I leave the rest to be stated by Dr. Gaston.

Notwithstanding the fact that calomel, in combination with quinine, had been given previously by Dr. Edwards, which I regarded as a judicious prescription for the control of the fever, we concluded to carry the mercurial treatment further, to combat the inflammatory tendencies in the case.

It was thought best also to limit the patient to a light diet for the present, until the febrile disturbance should be controlled, and chicken soup, with rice boiled in it, and tea with toast was recommended, with the addition afterwards of sweetmilk or buttermilk as he might prefer.

After administering three doses of 2 grains of calomel and 10 grains of bichlorate of soda, with intervals of two hours, during the latter part of the night, the bowels were not moved and the patient took 2 tablespoonsful of castor oil with a teaspoonful of spirits of turpentine at one dose, during the morning of the 26th, which brought away copious fecal evacuations in the course of the day.

On the 27th the patient took at intervals of three hours, sulphate of quinine, grs. iij; and camphorated Dover's powders, grs. ij. This was continued also on the following day.

In the meantime local applications were made constantly over the right lumbar and iliac regions by the following mixture of belladonna ointment, ℥ j; and camphor, ʒ i; rubbed in with the hand and afterwards covered with flannel.

The patient took another dose of castor oil on the evening of of the 28th of June, which had its cathartic effect early next morning most satisfactorily.

June 29th, I saw the patient again in consultation with Dr. Edwards and finding the inflammatory developments then well defined in the right lumbar and iliac regions, with acute sensibility on palpation within the crest of ilium, and without any diffused abdominal tenderness or distention, it became evident that we had to deal with a case of acute psoas abscess.

The febrile symptoms had diminished with the localization of the inflammation, and the pulse counted 110, while the temperature was 101°. Being desirous of lessening the extent of the suppuration, if it could not be arrested, I recommended to use internally, infusion of sepentaria, Oj; with chlorate of potash, ʒ j; in doses of 2 tablespoonsful every two hours. In the meantime o apply externally, every three hours, over the inflammation, mercurial and belladonna oint., each ℥ ss; iodine, 5 grains; iodide of potash, 30 grains. On this. a fresh poultice of flax seed and hops was directed to be kept up with regularity every three hours.

The diet was recommended to be of all such articles as were nutritious and easily digested, leaving the patient to his choice, as he had been using during the past few days but little solid food.

As heretofore, an occasional spoonful of castor oil was taken, bringing away always profuse fæcel discharges, indicating that the whole alimentary canal was performing its functions in a satisfactory manner.

In the meantime, it was thought that the comfort of the patient would be promoted by changing his quarters, and he was transferred to another boarding-house without in anyway aggravating his condition, though with some inconvenience on account of the sensitiveness in the structures of the lumbar and iliac regions under movements of the body even when in bed.

It was not attended with such an exacerbation as had followed his former removal from one room to another, and yet the indications of local inflammation had greatly increased.

July 1, a relative of the patient called at my office requesting me to visit him and take charge of the case, as he had ascertained that this would be satisfactory to Dr. Edwards, to which I replied that it was not in accordance with my sense of propriety to assume the management of the patient except in connection with the attending physician, and it was understood that he would be notified to meet me at an appointed hour.

At that time we instituted a careful examination of the whole abdominal region, and found that the patient could not rest upon his left side without great discomfort throughout the right lumbar and iliac regions, but on being placed in a sitting position a well-marked prominence was observed immediately in front of the right kidney and extending forward above the crest of the ilium into the iliac region. Dr. Edwards and myself found dullness on percussion over this prominence and a sense of deep fluctuation upon palpation, while there was great tenderness on pressure from the border of the sacro-lumbalis muscle, around above the crest of the ilium to the iliac region. With those indications of a deep psoas abscess, it was expected that poulticing for twenty-four hours would lessen the intervening tissue so as to reach the pus more readily by an incision, and directions were given accordingly. He continued to take the infusion of serpentaria with chlorate of potash, and was directed ¼ grain of morphine every four hours until relieved of pain or he should sleep at night. The febrile excitement alternating with chilliness had notably diminished at this time.

Drs. Edwards and Bosworth, with the relative of the patient, met me at his room on July 2. The patient was comparatively free from suffering, and upon examination of the local trouble, the prominence had disappeared, while there was less sensitiveness upon pressure and no evidence of fluctuation where it had previously existed. Instead, therefore, of proceeding to make an incision, I determined simply to make an exploratory puncture, and upon introducing the needle of my aspirator at the most salient lateral aspect of the right lumbar region, about midway between the cartilage of the ribs and the crest of the ilium, it was passed obliquely backwards through a dense membrane into a cavity, without any discharge in drawing back the piston. Through the same opening in the skin the trocar was then passed obliquely forward, again encountering a dense membrane which afforded considerable resistance before it entered an open space, admitting of free movement. There was no discharge indicating the presence of anything in this space, which was evidently a cavity surrounded by a pyogenic membrane, the contents of which had been evacuated internally. I had stated before making this exploratory puncture that in view of the subsidence of the protruberance and the disappearance of the fluctuation, it was probable that there was nothing to remove; and now gave it as my conclusion that the abscess that had existed on the day previous, found an outlet through the wall of the colon where it lies in contact with the quadratus lumborum and psoas muscles, without the intervention of peritonium.

Being called up by telephone after midnight to see the patient with Dr. Edwards, he was found with legs and feet cool, and suffering from nervousness, for which a mixture of camphor water and Hoffman's anodyne was given with mustard plasters to his ankles.

July 3. There was reported this morning a good result of the above treatment, with sleep during the subsequent hours until after daylight. There had been free evacuations from the bowels early in the morning, and as it was requested to save them for inspection, a portion of the fecal matter of apple-butter or mushy consistence, having a yellow drab hue, was preserved, which filled about one half the capacity of an ordinary chamber pot. We were told that at least a similar quantity had been thrown out presenting very similar appearance. While the fecal mass was for the most part of a homogeneous consistency, there were portions of a different color interspersed with it, which satisfied Dr. Edwards and myself of the presence of pus, thus confirming the impression that an abscess had discharged into the ascending colon. The nervous disturbance of the night previous passed off completely with these copious discharges of fecal matter and pus, which could not have been less than a half gallon, from the statement made by his relative who occupied the room with the patient. It is unfortunate that there was no microscopic or other process instituted for the detection of the pus in these evacuations; but as far as macroscopic examinations can be relied upon in connection with the history of the case, there could be no doubt as to the evacuation of a psoas abscess through the colon. The pulse and temperature were nearly normal and the flushes of heat and chilliness had ceased entirely, so that complete relief was anticipated.

July 4th. Not having seen the patient during the day on account of absence from the city, I found a note from his relative upon my slate on returning to my office in the evening, requesting me to visit him. Dr. Edwards met me at the room of the patient, and we learned that he had been out of bed, sitting up in a chair on two occasions during the day, writing letters, contrary to our expectations, and that he was feeling badly since, doubtless, from over.exertion. As there had been no further discharges from the bowels he was directed to take a grain of calomel every four hours until a movement should occur, when it should be suspended. He was also ordered five grains of quinine night and morning, with a continuation of the infusion of serpentaria and chlorate of potash every three or four hours.

July 5th. Upon seeing him together at 8 a. m., I learned from Dr. Edwards that the bowels commenced movement after the first dose of the calomel, and being moved freely several times before midnight, a teaspoonful of elixir of paregoric had been given to check the action, with the desired effect. He had passed the rest of the night quietly, and there had only been a single evacuation this morning. These discharges were reported to be consistent fecal matter of the same aspect as those previously noted, but had not been kept for an inspection. The indications of local trouble had not ceased, and there was a perceptible induration along the sheath of the psoas muscle reaching down towards the inguinal region, which presented soreness. There was not, however, any tenderness upon pressure, over the abdomen, nor any tympanitis, while the previous constitutional disturbance had disappeared, and the trouble was local. This induced a reapplication of the compound ointment already prescribed, with the use of poultices extending over the lumbar and iliac regions.

At our evening visit he was given another grain of calomel and 5 grains of quinine with instructions to use the camphor water and Hoffman's anodyne, if he did not rest, with the injunction to give ¼ grain sulph. morphine to procure sleep if required.

July 6. At 8 a. m. Dr. Edwards and I saw the patient and found that he had passed a comfortable night, feeling refreshed, and had enjoyed his breakfast. Finding the sensitiveness less over the tract of inflammation, the ointment was discontinued, with directions to keep up a light flaxseed meal and hop poultice, over the lumbar and iliac region. A nutritious diet was recommended, with small doses of quinine night and morning, and the use of milk punch as often as desired.

In the course of the day I learned that the services of Dr. Edwards and myself were not required any further, and I leave the sequel to be reported subsequently, if the circumstances warrant any further comment.

